# The Prognostic Value of Tumor HER2 Expression in Predicting Oncological Outcomes of Patients with Advanced Urothelial Carcinoma: A Systematic Review and Meta-Analysis

**DOI:** 10.3390/ijms27094130

**Published:** 2026-05-05

**Authors:** Mohammad Hossein Alibeiginejad, Alireza Esmaielpour, Ichiro Tsuboi, Akihiro Matsukawa, Takafumi Yanagisawa, Keiichiro Mori, Mehdi Kardoust Parizi

**Affiliations:** 1 Department of Urology, Shariati Hospital, Tehran University of Medical Sciences, Tehran 14117-13135, Iran; tums1998@gmail.com (M.H.A.); aesmailpoor0@gmail.com (A.E.); 2 Department of Urology, Okayama University Graduate School of Medicine, Dentistry and Pharmaceutical Sciences, Okayama 700-8558, Japan; 3 The Jikei University School of Medicine, Tokyo 105-8461, Japan; 4 Department of Urology, Comprehensive Cancer Center, Medical University of Vienna, 1090 Vienna, Austria; 5 Uro-Oncology Research Center, Tehran University of Medical Sciences, Tehran 14197-33141, Iran

**Keywords:** human epidermal growth factor receptor 2, HER2, urothelial carcinoma, prognosis metastatic, advanced

## Abstract

The biologic and prognostic value of tumor human epidermal growth factor receptor 2 (HER2) expression in patients with advanced urothelial carcinoma (UC) who undergo systemic therapies remains controversial. A systematic search of English-language literature using PubMed, Scopus, and Cochrane Library was performed in October 2025 according to the preferred reporting items for systematic reviews and meta-analyses (PRISMA) protocol to evaluate the prognostic value of tumor HER2 expression in predicting oncological outcomes of patients with advanced UC. The primary endpoints were recurrence-free survival (RFS), progression-free survival (PFS), cancer-specific survival (CSS), and overall survival (OS). Seventeen studies comprising 4909 patients were eligible. HER2 expression was significantly associated with inferior RFS (HR 1.68, 95% CI 1.16–2.42; *p* = 0.005) and CSS (HR 1.36, 95% CI 1.10–1.68; *p* = 0.004), but not with OS (HR 1.03, 95% CI 0.54–1.80) or PFS (HR 0.97, 95% CI 0.50–1.89), in patients with advanced UC. In conclusion, tumor HER2 expression may identify a subgroup of patients with advanced UC at increased risk of recurrence and cancer-specific mortality, supporting its potential role as a prognostic biomarker. However, standardized assessment and prospective studies are warranted to define its utility for risk stratification and therapeutic targeting.

## 1. Introduction

Despite significant progress in the field of systemic treatment strategies, including the introduction of immune checkpoint inhibitors that have modestly improved outcomes in recent years [1], locally advanced or metastatic urothelial carcinoma (UC) remains a major challenge in oncology due to its aggressive nature and poor prognosis. The survival rates for patients with advanced UC are dismal, with the 5-year survival rate reported to be less than 5%, highlighting the urgent need for novel therapeutic approaches [2,3].

For more than two decades, cisplatin-based chemotherapy represented the standard first-line treatment for metastatic UC. This paradigm has recently been reshaped by the advent of immune checkpoint inhibitors and practice-changing randomized trials demonstrating superior overall survival compared with platinum-based chemotherapy, highlighting the critical need to identify novel biomarkers and molecular pathways involved in UC tumorigenesis. These therapies, including pembrolizumab, atezolizumab, and nivolumab, have revolutionized clinical practice by offering an alternative for patients who are not candidates for cisplatin therapy due to comorbidities or prior chemotherapy failures [4]. Moreover, the encouraging results from these trials emphasize the importance of further exploring the molecular underpinnings of UC tumorigenesis, as this could reveal new targets for therapy and lead to the identification of predictive biomarkers for response to treatment. The introduction of immunotherapy into clinical practice also highlights the need for a more refined approach to patient stratification based on molecular characteristics [5].

As UC is a heterogeneous disease with varying clinical presentations and molecular profiles, there is a growing recognition that personalized treatment strategies, guided by the tumor’s molecular characteristics, will lead to better outcomes. These biomarkers not only hold potential for better diagnosis and prognosis but can also provide valuable insights into the mechanisms of resistance to current treatments, helping to inform the development of more effective therapeutic strategies [6,7].

One promising avenue of research in this area is the exploration of the human epidermal growth factor receptor 2 (HER2), a member of the ErbB family of receptor tyrosine kinases, which has been implicated in the pathogenesis of several cancers, including UC. HER2 has garnered significant attention as a prognostic and predictive biomarker across various cancers due to its critical role in tumorigenesis. Overexpression or gene amplification of HER2 is often associated with poor prognosis and has been linked to aggressive disease behavior and resistance to conventional therapies in certain cancers, such as breast and gastric cancer [8,9].

In UC, however, the role of HER2 as a biomarker remains controversial. While some studies suggest that HER2 overexpression or amplification may correlate with poor outcomes, other studies have failed to establish a clear prognostic or predictive value for HER2 in UC [7,9].

This discrepancy may be attributed to variations in study designs, patient populations, and assessment methods, which underscores the importance of standardizing HER2 testing in UC and establishing a robust framework for interpreting its clinical significance. The conflicting results regarding the role of HER2 in UC highlight the challenges in translating molecular findings into clinically actionable information.

A key issue is the lack of consensus on the optimal method for assessing HER2 expression and amplification in UC tissues. Immunohistochemistry (IHC) and fluorescence in situ hybridization (FISH) are commonly used techniques, but there is no standardized threshold for determining HER2 positivity, and results can vary depending on the laboratory or institution performing the test. Additionally, HER2 testing in UC is complicated by tumor heterogeneity, where different regions of the tumor may exhibit varying levels of HER2 expression, further complicating the interpretation of results [10]. Therefore, improving the accuracy and reproducibility of HER2 testing in UC is crucial for determining its potential clinical utility as a biomarker for prognosis and therapy selection.

Despite these challenges, the potential of HER2 as a therapeutic target in UC remains an area of intense investigation. Targeted therapies, such as trastuzumab and pertuzumab, which have shown efficacy in HER2-positive breast cancer, are being explored in clinical trials for UC. In fact, early-phase studies have demonstrated promising results for the use of HER2-targeted agents in UC patients who exhibit HER2 overexpression or amplification, particularly when combined with chemotherapy or immunotherapy [11]. These findings suggest that HER2 could become an important biomarker for selecting patients who are likely to benefit from targeted therapies, providing a more personalized treatment approach for individuals with advanced UC.

In addition to HER2, other molecular pathways and biomarkers are being investigated to improve the understanding of UC’s complex biology and to identify potential therapeutic targets. For instance, alterations in the FGFR3 (fibroblast growth factor receptor 3) gene and mutations in the TP53 (tumor protein p53) gene have been implicated in UC pathogenesis and could serve as additional targets for treatment [6,12]. Moreover, the tumor microenvironment, including immune checkpoint molecules such as PD-L1, has emerged as an important area of research in UC, with several studies exploring the role of immune modulators in enhancing the anti-tumor immune response. As research into these molecular targets progresses, it is likely that the development of multi-targeted therapies will become increasingly important in the treatment of advanced UC [13].

To better understand the prognostic significance of HER2 in UC, we conducted a systematic review to synthesize the available evidence on the association between HER2 expression and oncological outcomes in patients with advanced UC. Our aim was to provide a comprehensive overview of the current state of the literature, highlight the strengths and limitations of existing studies, and identify gaps in knowledge that could inform future research efforts. By focusing on the prognostic role of HER2, we hope to contribute to the ongoing efforts to improve the management of UC through the identification of reliable biomarkers that can guide treatment decisions and ultimately improve patient outcomes.

## 2. Results

### 2.1. Literature Search Process

**Figure 1:**
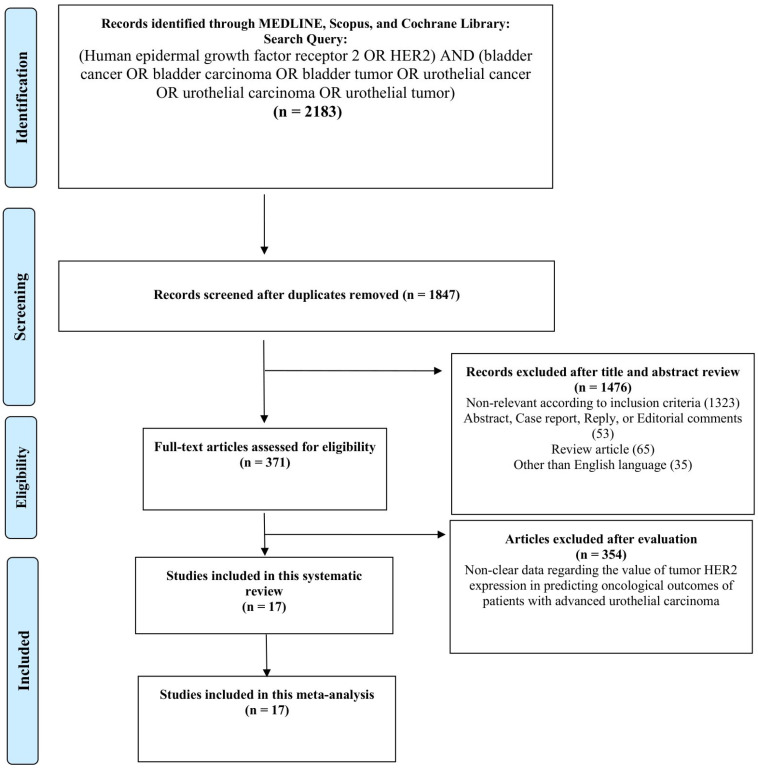
PRISMA flow chart for article selection process to analyze the value of tumor HER2 expression in predicting oncological outcomes of patients with advanced urothelial carcinoma.

A total of 2183 studies were identified by our initial literature search and 336 duplicates were removed. Then, 1476 and 354 articles were excluded after title/abstract evaluation and full-text assessment, respectively. Finally, 17 were included for qualitative and quantitative evidence synthesis (Figure 1).

### 2.2. Characteristics of the Included Studies and Patients

The characteristics of the studies and the clinical data are described in Table 1 and Table 2. These studies were published between 2002 and 2024 and included 4909 patients with advanced or metastatic UC. There were nine studies from Asia [14,15,16,17,18,19,20,21,22], four from Europe [23,24,25,26], two from North America [27,28], and two from the Europe/North America region [29,30].

### 2.3. Risk of Bias Assessment (RoB)

Authors’ assessments of each domain for every study included are depicted in Supplementary Table S4. We identified a moderate overall RoB in the majority of the included studies.

### 2.4. Meta-Analysis

#### 2.4.1. Tumor HER2 Expression and Overall Survival (OS)

**Figure 2:**
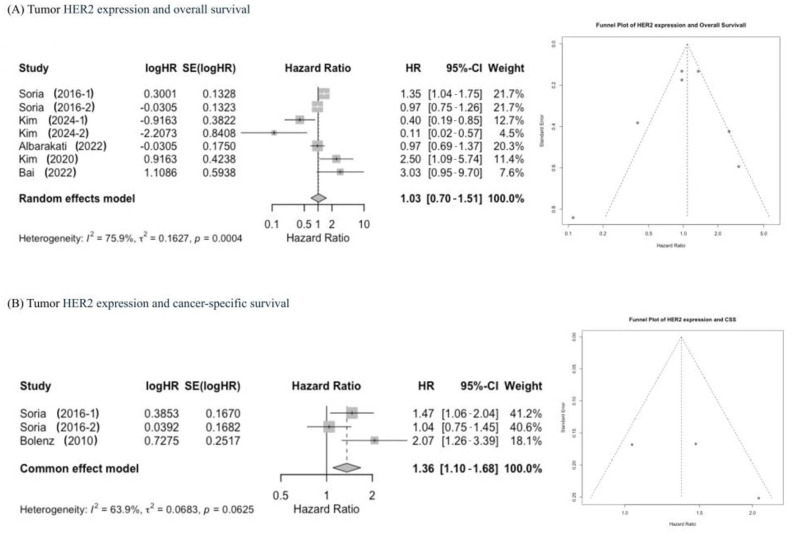

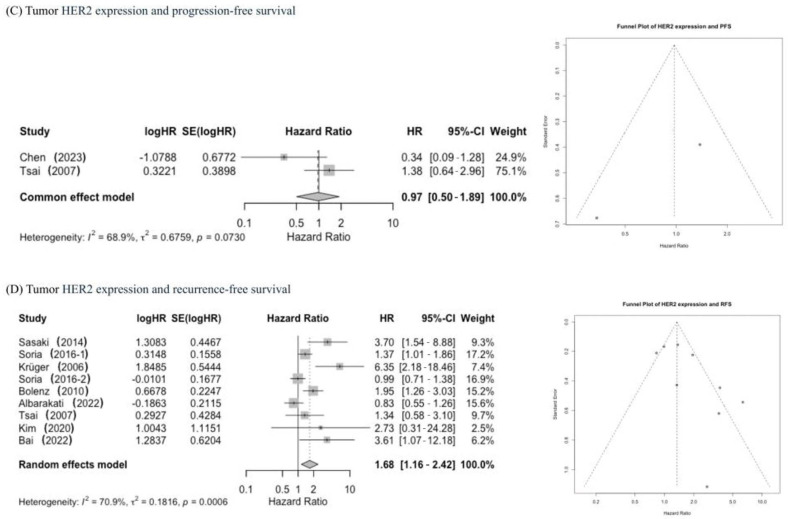
Forest plots and funnel plots showing the pooled hazard ratio showing association between tumor HER2 expression and oncological outcomes. CI: confidence interval, HR: hazard ratio. Described studies cited from Refs. [14,15,18,19,20,21,26,27,28,29,30].

The impact of tumor HER2 expression on OS was investigated in seven studies [18,20,21,28,29,30], with a total of 1826 patients. Tumor HER2 expression was not associated with OS (pooled HR 1.03, 95% CI: 0.70–1.51) A statistically significant heterogeneity was found among included studies using the Chi-square and I2 tests (I^2^ = 75.9%, *p* = 0.0004); the weights were from a random effect model to analyze pooled HR. Funnel plots identified four studies over the pseudo 95% CI (Figure 2A).

#### 2.4.2. Tumor HER2 Expression and Cancer-Specific Survival (CSS)

The impact of tumor HER2 expression on CSS was investigated in three studies [27,29,30], with a total of 1284 patients. Tumor HER2 expression was associated with worse CSS (pooled HR 1.36, 95% CI: 1.10–1.68). No statistically significant heterogeneity was found among included studies using the Chi-square and I2 tests (I^2^ = 63.9%, *p* = 0.0683); the weights were from a common effect model to analyze pooled HR. Funnel plots identified no study over the pseudo 95% CI (Figure 2B).

#### 2.4.3. Tumor HER2 Expression and Progression-Free Survival (PFS)

The impact of tumor HER2 expression on PFS was investigated in two studies [15,19], with a total of 150 patients. Tumor HER2 expression was not associated with PFS (pooled HR 0.97, 95% CI: 0.50–1.89). No statistically significant heterogeneity was found among included studies using the Chi-square and I2 tests (I^2^ = 68.9%, *p* = 0.0730); the weights were from a common effect model to analyze pooled HR. Funnel plots identified no study over the pseudo 95% CI (Figure 2C).

#### 2.4.4. Tumor HER2 Expression and Recurrence-Free Survival (RFS)

The impact of tumor HER2 expression on RFS was investigated in nine studies [14,19,20,21,26,27,28,29,30], with a total of 2319 patients. Tumor HER2 expression was associated with worse RFS (pooled HR 1.68, 95% CI: 1.16–2.42). Statistically significant heterogeneity was found among included studies using the Chi-square and I2 tests (I^2^ = 70.9%, *p* = 0.0006); the weights were from a random effect model to analyze pooled HR. Funnel plots identified three studies over the pseudo 95% CI (Figure 2D).

## 3. Discussion

In this systematic review and meta-analysis, we found that tumor HER2 expression is significantly associated with inferior RFS and CSS, whereas no significant association was observed with OS or PFS. These results suggest that tumor HER2 expression may reflect a more aggressive tumor biology in advanced UC, particularly with respect to recurrence risk and disease-specific mortality. The association between tumor HER2 expression and worse RFS observed in our analysis supports the hypothesis that HER2-driven signaling contributes to tumor aggressiveness, early relapse, and resistance to conventional therapies [9].

HER2 overexpression has been linked to enhanced cell proliferation, invasion, angiogenesis, and metastatic potential in several solid tumors, most notably breast and gastric cancers [31]. Similar biological mechanisms may underlie disease recurrence in UC, where HER2-positive tumors could represent a molecularly distinct and clinically unfavorable subgroup. In UC, HER2 alterations are more heterogeneous than in breast or gastric cancers, encompassing not only protein overexpression but also ERBB2 amplification and activating mutations [32]. Genomic profiling studies have demonstrated ERBB2 amplification in a subset of muscle-invasive and advanced UC, frequently co-occurring with chromosomal instability and luminal molecular subtypes. This genomic context may partly explain the association between HER2 expression and aggressive tumor behavior, as ERBB2-driven tumors often exhibit enhanced proliferative capacity and survival signaling [33].

A large-scale pathological analysis by Laé and colleagues provided one of the most robust assessments of HER2 status in invasive UC using standardized criteria analogous to those established in breast cancer. In this study of over 1000 patients, HER2 protein expression was systematically evaluated by IHC, with equivocal and positive cases undergoing confirmatory FISH [34]. The authors reported HER2 overexpression in approximately 9% of tumors, while true ERBB2 gene amplification was identified in just over 5% of cases, underscoring that only a subset of HER2-expressing tumors harbor genomic amplification. Importantly, all tumors with strong (3+) HER2 expression demonstrated gene amplification, whereas none of the equivocal (2+) cases were amplified, highlighting a high concordance between intense protein overexpression and underlying genomic alteration. This finding supports the biological relevance of high-level HER2 expression in UC and reinforces the importance of confirmatory molecular testing in equivocal cases. A particularly notable observation was the high prevalence of intratumoral heterogeneity, detected in more than one-third of HER2-positive tumors, with sharply demarcated regions of HER2-amplified and non-amplified cells within the same lesion [34]. Such heterogeneity has significant clinical implications, as it may contribute to sampling bias, misclassification of HER2 status in small biopsy specimens, and variable therapeutic responses.

The prognostic relevance of HER2 in UC was further elucidated by a detailed pathological study assessing HER2 status in matched primary bladder tumors and corresponding lymph node metastases [35]. In this cohort of patients with clinically node-negative disease who were found to have nodal involvement at cystectomy, HER2 gene amplification was significantly more frequent in lymph node metastases than in primary tumors, highlighting the enrichment of HER2-altered clones during metastatic progression. Importantly, HER2 amplification demonstrated a high degree of concordance between primary tumors and their matched metastases, indicating that ERBB2 amplification represents a stable genomic event rather than a transient or stochastic alteration. In contrast, HER2 protein expression assessed by IHC showed greater variability and poorer concordance, particularly when compared with gene amplification status, underscoring the limitations of IHC-based assessment alone. From a clinical standpoint, HER2 amplification in the primary tumor was significantly associated with inferior OS, whereas IHC-defined HER2 expression failed to predict an outcome. These findings provide strong biological support for the results of our meta-analysis, which identified HER2 positivity as a marker of aggressive disease biology, particularly with respect to recurrence and cancer-specific mortality. The enrichment of HER2 amplification in metastatic lesions offers a plausible mechanistic explanation for the higher recurrence risk observed in HER2-positive tumors, suggesting that HER2-driven clones may preferentially seed and expand within metastatic niches. Moreover, the superior prognostic performance of FISH compared with IHC in this study aligns with the methodological heterogeneity and variable prognostic associations observed across the studies included in our analysis. These data reinforce the concept that HER2 amplification, rather than protein expression alone, may be the most biologically and clinically relevant alteration in advanced UC.

Collectively, these results provide a compelling explanation for the inconsistent prognostic associations reported across earlier studies and align with the heterogeneity observed in our meta-analysis. By demonstrating that a minority but clinically meaningful proportion of invasive bladder carcinomas harbor bona fide ERBB2 amplification, this study laid essential groundwork for the rational development of HER2-targeted therapies in UC. Moreover, it supports the concept that HER2-positive UC represents a distinct biological subgroup that may benefit from molecularly guided treatment strategies, particularly in the setting of locally advanced or metastatic disease.

HER2 activation in UC promotes oncogenesis through engagement of key downstream pathways, including PI3K/AKT/mTOR and MAPK/ERK signaling, which regulate cell cycle progression, apoptosis resistance, and metabolic adaptation. Crosstalk between HER2 and other receptor tyrosine kinases, such as EGFR and FGFR3, has also been reported, potentially amplifying oncogenic signaling and contributing to intratumoral heterogeneity. Importantly, alterations in downstream effectors such as PIK3CA mutations or PTEN loss may modulate HER2 signaling output, influencing both tumor aggressiveness and responsiveness to therapy [36]. These molecular interactions provide a plausible mechanistic basis for the observed association between HER2 expression and poorer recurrence-related outcomes, particularly in the context of treatment resistance and early disease relapse.

A major advance in understanding HER2 biology in UC emerged from comprehensive genomic analyses, particularly the work of Choi et al., who performed transcriptomic profiling of muscle-invasive bladder cancer and identified distinct molecular subtypes with divergent clinical behavior [37]. In this study, ERBB2 amplification and HER2 overexpression were enriched within luminal subtypes, which exhibited activation of receptor tyrosine kinase signaling and differential sensitivity to targeted therapies. Importantly, while luminal tumors were initially thought to confer a more favorable prognosis, subsequent analyses revealed that HER2-positive luminal tumors represent a biologically aggressive subset characterized by increased proliferation and genomic instability. Choi and colleagues demonstrated that ERBB2-driven signaling intersected with PI3K/AKT and MAPK pathways, reinforcing mechanistic links between HER2 activation and aggressive tumor phenotypes [37]. These molecular characteristics provide a compelling biological explanation for the significant associations between HER2 expression and inferior RFS and CSS observed in our meta-analysis, suggesting that HER2 positivity identifies a subset of tumors with an inherently higher propensity for recurrence and disease-specific mortality. Rather than being uniformly prognostic across all UC cases, HER2 expression likely exerts its clinical impact within specific molecular ecosystems, modulated by co-occurring alterations and pathway dependencies. This context-dependent effect is consistent with our findings of endpoint-specific prognostic relevance, with stronger associations for recurrence-related and cancer-specific outcomes than for OS. This subtype-specific framework may partly explain inconsistencies across prior prognostic studies and highlights the importance of integrating HER2 status with broader molecular classification systems. From a translational perspective, the work of Choi et al. supports the rationale for biology-driven patient selection in HER2-targeted clinical trials and underscores the limitations of relying solely on immunohistochemical expression without molecular context.

Beyond its role in tumor cell intrinsic signaling, HER2 may also influence the tumor microenvironment in UC. Preclinical data suggest that HER2-driven tumors can exhibit increased angiogenic signaling and altered immune contexture, including modulation of cytokine production and immune cell infiltration [38]. Such effects may contribute to immune evasion and reduced sensitivity to conventional chemotherapy. Moreover, emerging evidence indicates that HER2 expression may interact with immune checkpoint pathways, raising the possibility that HER2 status could influence response to immunotherapy or inform rational combination strategies [39].

The consistent association with poorer CSS further reinforces the relevance of HER2 as a marker of aggressive disease biology rather than a nonspecific correlate of patient survival. In contrast, HER2 expression was not associated with OS or PFS. Several factors may explain these findings. OS is influenced by multiple downstream variables, including subsequent lines of therapy, patient comorbidities, and competing causes of death, which may dilute the prognostic impact of a single biomarker. In addition, the increasing use of immune checkpoint inhibitors and other systemic agents in later lines of treatment could mitigate the effect of HER2 status on OS [2,40]. Similarly, the lack of association with PFS should be interpreted cautiously, given that only two studies with a limited sample size contributed to this analysis, resulting in reduced statistical power. Our results align with prior individual studies reporting an association between HER2 expression and adverse pathological features, higher tumor grade, and advanced stage in UC, while also helping to reconcile conflicting evidence regarding survival outcomes. Importantly, the prognostic value of HER2 appears to be endpoint-specific, with stronger associations observed for recurrence and cancer-specific mortality than for broader survival metrics. This distinction highlights the need to carefully select clinically meaningful endpoints when evaluating molecular biomarkers in advanced UC. Notably, substantial heterogeneity was observed in several analyses, particularly for OS and RFS. This heterogeneity likely reflects methodological differences across studies, including variability in patient populations (locally advanced vs. metastatic disease), treatment regimens, follow-up duration, and, critically, HER2 assessment techniques. HER2 positivity was defined using heterogeneous immunohistochemical scoring systems, different antibodies, variable cut-off thresholds, and inconsistent use of confirmatory FISH testing. In breast cancer, HER2 IHC interpretation is based on complete, circumferential membrane staining and percentage-based cut-offs. In contrast, gastric cancer uses a modified approach in which strong but non-circumferential staining patterns, such as basolateral or lateral membrane reactivity, may qualify as positive. For gastric biopsies, the usual percentage requirement applied in breast pathology is not used; instead, evaluation is allowed when a small cluster of clearly identifiable tumor cells shows true membranous staining. The assessment of staining intensity in gastric cancer also relies more heavily on the level of magnification needed to appreciate membrane positivity, with strong reactions apparent at low power and weaker categories requiring progressively higher magnification. These adaptations reflect the greater heterogeneity and distinct staining behavior seen in gastric tumors [41]. Unlike breast and gastric cancers, where standardized HER2 testing algorithms are well established, no universally accepted framework exists for UC, limiting comparability across studies and potentially attenuating observed associations [31]. Beyond molecular prognostic markers such as HER2, imaging-based approaches have also been explored to refine risk assessment in bladder cancer. For example, a recent study suggested that FDG PET/CT may improve detection of occult lymph node involvement in patients with bladder cancer with variant histology compared with conventional imaging, underscoring the complementary role of imaging in disease staging and prognostication [42].

Our results suggest that HER2 expression may help identify a subgroup of patients at higher risk of recurrence and disease-specific death who could potentially benefit from intensified surveillance strategies or HER2-targeted approaches. However, the lack of association with OS underscores the need for prospective validation and predictive biomarker analyses within clinical trials.

From a therapeutic perspective, advances in HER2-targeted strategies have expanded beyond traditional monoclonal antibodies to include antibody–drug conjugates (ADCs), bispecific antibodies, and small-molecule tyrosine kinase inhibitors [43,44]. ADCs targeting HER2 are of particular relevance in UC, as they may overcome limitations related to heterogeneous HER2 expression by leveraging bystander killing effects. Early clinical studies have demonstrated meaningful activity of HER2-directed ADCs in patients with HER2-expressing UC, including those with low or intermediate expression levels [45].

The therapeutic relevance of HER2 expression in UC has been substantially strengthened by the phase II clinical evaluation of disitamab vedotin (DV, RC48-ADC), a novel HER2-directed antibody–drug conjugate, in patients with HER2-positive locally advanced or metastatic disease who had progressed after standard systemic therapies [11]. In two multicenter, open-label phase II studies enrolling over 100 heavily pretreated patients, DV demonstrated a high objective response rate exceeding 50%, with durable responses and clinically meaningful progression-free and overall survival outcomes. Importantly, treatment activity was consistently observed across clinically challenging subgroups, including patients with visceral metastases and those previously treated with immune checkpoint inhibitors, underscoring the robustness of HER2 as a therapeutically actionable target in advanced UC. These results are particularly notable given the aggressive disease biology and limited treatment options in this patient population. From a biological and clinical standpoint, the efficacy of DV directly aligns with the findings of our meta-analysis, which identifies HER2 expression as a marker of increased recurrence risk and cancer-specific mortality. While HER2 positivity in our pooled analysis was associated with inferior recurrence-related outcomes, the favorable responses observed with HER2-targeted therapy suggest that this adverse prognostic subgroup may derive disproportionate benefit from biomarker-guided treatment strategies. Furthermore, the demonstration of therapeutic activity across both IHC 2+ and 3+ tumors highlights the clinical relevance of HER2 heterogeneity and supports emerging concepts of HER2-low disease in UC. The manageable safety profile of DV, despite a substantial incidence of treatment-related adverse events, further supports its feasibility in heavily pretreated patients.

Building upon the encouraging results of the phase II studies, a subsequent phase III randomized trial further validated the clinical relevance of HER2-targeted therapy in UC [46]. In this study, the combination of disitamab vedotin and the anti-PD-1 antibody toripalimab was compared with standard platinum-based chemotherapy in previously untreated patients with HER2-expressing locally advanced or metastatic disease. The combination regimen demonstrated significant improvements in key clinical outcomes, including progression-free survival (13.1 vs. 6.5 months) and OS (31.5 vs. 16.9 months), along with a higher objective response rate (76.1% vs. 50.2%). Notably, therapeutic benefit was observed across a broader spectrum of HER2 expression, including tumors with lower HER2 levels, further supporting the expanding clinical relevance of HER2-directed strategies in UC.

Another therapeutic agent Trastuzumab deruxtecan (T-Dxd), a topoisomerase 1 inhibitor, is a HER-2-binding ADC previously approved for breast carcinoma. The DESTINY-PanTumor02 Phase II Trial included a urothelial cancer cohort with disease progression post at least one prior standard agent and required tumors having HER-2-overexpression by IHC (3+ or 2+). In the 41 patients, the Objective Response Rate of T-Dxd was 56% for HER2 (3+ by IHC score). The total PFS was 7 months, and there was a combined OS of 12.8 months in this cohort [47].

Collectively, these data provide critical translational validation that HER2 in UC is not merely a prognostic indicator of aggressive tumor behavior, as observed in our meta-analysis, but also a predictive biomarker capable of guiding effective targeted therapy. This convergence of prognostic and therapeutic evidence underscores the importance of standardized HER2 assessment and supports the integration of HER2-directed agents into prospective treatment algorithms for advanced urothelial carcinoma.

These developments underscore the importance of refining molecular characterization of HER2 alterations; encompassing expression, amplification, and mutation status to optimize patient selection and maximize therapeutic benefit. In this context, integrating molecular profiling with standardized HER2 assessment may be critical for translating the prognostic relevance of HER2 into effective, biology-driven treatment strategies in advanced UC.

Our study is not without limitations. First, most of the included studies were retrospective in nature, carrying an inherent risk of bias. Although the overall risk of bias was moderate, residual confounding cannot be entirely excluded. Second, the relatively small number of studies reporting CSS and PFS limited the robustness of these specific analyses and may have affected the strength of the conclusions. Third, the lack of patient-level data precluded detailed subgroup analyses according to treatment modality, disease burden, or molecular subtype, which may be clinically relevant modifiers of prognosis. Fourth, variability in pre-analytic conditions (e.g., tissue age, fixation parameters, ischemia time, and specimen handling) across studies should be considered, as these factors may influence HER2 assessment and limit the interpretation and comparability of the findings. Finally, although formal funnel plot assessments did not demonstrate major asymmetry for most endpoints, the possibility of publication bias cannot be completely ruled out. Despite these limitations, this meta-analysis represents one of the most comprehensive assessments to date of the prognostic value of HER2 expression in advanced urothelial carcinoma. By integrating evidence across multiple oncological outcomes, our findings provide a clearer and more nuanced understanding of the clinical relevance of HER2 in this disease and highlight areas for future research.

## 4. Materials and Methods

### 4.1. Search Strategy

This systematic review and meta-analysis was conducted in accordance with the Preferred Reporting Items for Systematic Reviews and Meta-analyses (PRISMA) statement and AMSTAR2 checklist (Supplementary Section S1, Supplementary Tables S1, S2, and S3) [48,49]. A comprehensive literature search was performed across PubMed, Scopus, and Cochrane Library by two independent authors in October 2025, to find studies assessing the prognostic value of tumor HER2 expression in predicting oncological outcomes of patients with advanced UC. After the primary screening based on study title and abstract, all full-text papers were assessed and excluded with reasons. Any discrepancies were resolved by referring to the senior author. The following search string was used: (human epidermal growth factor receptor 2 OR HER2) AND (bladder cancer OR bladder carcinoma OR bladder tumor OR urothelial cancer OR urothelial carcinoma OR urothelial tumor). The protocol of this systematic review and meta-analysis was registered in the International Prospective Register of Systematic Reviews database (PROSPERO, CRD420251239416).

### 4.2. Inclusion and Exclusion Criteria

The population, intervention, comparator, outcome, and study design (PICOS) approach was used to define the eligibility criteria. Studies were selected when advanced or metastatic UC patients with advanced or metastatic UC with tumor HER2 expression (P: population) who underwent systemic treatments (intervention) (I: interventions) were compared with advanced or metastatic UC without tumor HER2 expression (C: comparators) in terms of oncological survival outcomes (O: outcomes) using prospective or retrospective studies (S: study design). We included histologically confirmed UC patients where HER2 expression was assessed by IHC, with or without confirmatory FISH, and which reported at least one oncological survival outcome, including OS, CSS, RFS, and PFS, showing hazard ratio (HR) or risk ratio (RR) throughout the multivariate cox regression analysis, ensuring adjustment for potential confounders.

We excluded conference abstracts, replies, editorial comments, review articles, and papers written in a non-English language. Discrepancies were resolved by consensus or consultation with the senior reviewer.

### 4.3. Data Extraction

The full text of relevant studies was evaluated by two independent authors. In case of more than one study of the same cohort, we included only the largest or most recent study. Data were extracted on study name, authors, publication year, study region, study design, number of patients, technique of HER2 assessment, cut-off criteria for HER2 positivity, oncological outcomes, and followup duration. Independent correlation of tumor HER2 expression with oncologic outcomes were retrieved.

### 4.4. Risk of Bias Assessment

We assessed the risk of bias of the included studies according to the revised Quality Assessment of Diagnostic Accuracy Studies tool (QUADAS-2) [50]. Each bias domain and overall risk of bias were judged as “low”, “high”, or “unclear” risk of bias. Disagreements were resolved by consensus or consultation with other authors.

### 4.5. Statistical Analyses

A common- or random-effect model was used to estimate HRs and 95% confidence interval (CI). Significant heterogeneity was indicated by *p*  <  0.05 in the Cochrane’s Q tests and a ratio of > 50% in I^2^ statistics. With no heterogeneity among selected studies, we considered common (fixed) effect models to calculate pooled HRs. Visual inspection of a funnel plot was carried out to identify publication bias in our meta-analysis. IHC and FISH based assessments were combined and analyzed in the meta-analysis. We performed statistical analyses using R version 4.0.3 (2020; R Foundation for Statistical Computing, Vienna, Austria). The statistical significance level was set at *p*  <  0.05.

## 5. Conclusions

Tumor HER2 expression is associated with an increased risk of recurrence and cancer-specific mortality in patients with advanced UC, supporting its potential role as a prognostic biomarker. Future prospective studies using standardized HER2 assessment methods and incorporating modern systemic therapies are essential to define its role in risk stratification and to clarify its utility as a therapeutic target in advanced UC. As HER2 has high intratumoral heterogeneity, clinicians may also consider multiple biopsies or prioritizing FISH in equivocal cases.

## Figures and Tables

**Table 1:** Characteristics of included studies reporting the prognostic value of tumor HER2 in predicting oncological outcomes of patients with advanced urothelial carcinoma.

Study	Year	Study Design	Recruitment Period	Pts ^a^	Region	Treatment, n (%)
Krüger [23]	2002	Retrospective	1990–1999	138	Europe	RC, NAC 10 (7%), ACT 35 (25%)
Simon [24]	2003	Retrospective	NA	1853	Europe	RC
Langner [25]	2005	Retrospective	1984–2002	53	Europe	RNU
Krüger [26]	2006	Retrospective	1990–2000	132	Europe	RC, ACT 30 (22%)
Tsai [19]	2007	Retrospective	1989–1998	114	Asia	RC. RNU, ACT 55 (48%)
Kolla [16]	2008	Retrospective	1999–2007	90	Asia	RC
Bolenz [27]	2010	Retrospective	1984–2002	198	North America	RC, ACT 60 (30%)
Sasaki [14]	2014	Retrospective	1996–2012	171	Asia	RNU
Soria [29]	2016	Retrospective	1990–2008	732	Europe and North America	RNU, ACT 71 (10%)
Soria [30]	2016	Retrospective	1988–2003	354	Europe and North America	RC, ACT 35 (10%)
Kim [20]	2020	Retrospective	1999–2014	97	Asia	RC, PORT 28 (29%)
Albarakati [28]	2022	Retrospective	1988–2013	413	North America	RC
Bai [21]	2022	Retrospective	2015–2020	108	Asia	RC, ACT 19 (17%)
Chen [15]	2023	Retrospective	2021 –2022	36	Asia	RC, RT 2 (5%), IO 18 (50%)
Zhou [17]	2023	Retrospective	2016–2021	284	Asia	RC, CT 278 (98%), IO 165 (58%)
Kim [18]	2024	Retrospective	2005–2013	61	Asia	RC
Huang [22]	2024	Retrospective	NA	75	Asia	RC

RC: radical cystectomy, RNU: radical nephroureterectomy, CT: chemotherapy, ACT: adjuvant chemotherapy, NAC: neoadjuvant chemotherapy, IO: immunotherapy, RT: radiotherapy, PORT: post-operative radiotherapy. a: patients with available survival data.

**Table 2:** Characteristics of included patients reporting the prognostic value of tumor HER2 in predicting oncological outcomes of patients with advanced urothelial carcinoma.

Study	Age, Year	Tumor HER2 Assessment Method	Cut-Off Value	Tumor HER2-Positive Expression, n (%)	OS	PFS	CSS	RFS	Follow-Up Period, Months
Krüger [23]	Median: 64	IHC + DISH	IHC 3+ OR IHC 2+ with HER2/CEP17 ratio > 2.2	23 (13.5)	NA	NA	NA	HR: 3.70, CI 1.54–8.87, *p* = 0.003	Up to165
Simon [24]	NA	IHC	IHC Score = 3+ (strong, complete membranous staining in >10% cells)	57 (41.3)	NA	NA	NA	RR: 2.22, CI 1.13–4.25, *p* = 0.020	Median: 45.5
Langner [25]	Median: 70.3	FISH + IHC	IHC: Score 2+ or 3+ considered positive (per HercepTest)	Amplification: 93 (6.3), IHC positivity (2+ or 3+): 514 (34.3)	NA	NA	RR: 1.02, CI 0.76–1.35, *p* = 0.942	NA	Median: 42
Krüger [26]	Median: 64	IHC + FISH + NGS	IHC 2+ or 3+ considered HER2-positive	28 (77.8)	NA	HR = 0.34, CI 0.09–1.28, *p* = 0.11	NA	NA	Up to 13
Tsai [19]	Median: 64	IHC	Score 2+ or 3+ (complete membranous staining ≥10% cells)	262 (35.8)	HR: 1.35 CI: (1.04–1.75) *p* = 0.02	NA	HR: 1.47, CI 1.06–2.04, *p* = 0.02	HR: 1.37, CI 1.01–1.86, *p* = 0.04	Median: 35
Kolla [16]	Mean: 58	IHC	Score 2+ or 3+ are HER2-positive	50 (55.6)	NA	NA	NA	RR: 2.51, CI 1.32–4.10, *p* = 0.016	Median: 46
Bolenz [27]	Median: 66.7	IHC	Score 3 (strong membranous staining)	23 (21)	NA	NA	NA	HR: 6.35, CI 2.19–18.5, *p* = 0.001	NA
Sasaki [14]	NA	IHC	Scores 2+ and 3+ considered “abnormal”	126 (36)	HR: 0.97 CI: (0.75–1.26) *p* = 0.8	NA	HR: 1.04, CI 0.75–1.45, *p* = 0.8	HR: 0.99, CI 0.71–1.37, *p* = 0.9	Median: 123
Soria [29]	Median: 69.8	IHC + FISH	IHC 2+ or 3+	125 (44)	NA	NA	NA	NA	Median: 63.5
Soria [30]	Median: 66.3	IHC	HER2 2+ or 3+ = HER2+	14 (22.9)	HR: 0.401 CI: (0.19–0.848) *p* = 0.017	NA	NA	NA	NA
Kim [20]	NA	IHC	HER2 2+ or 3+ = HER2+	8 (24.2)	HR: 0.113 CI: (0.024–0.536) *p* = 0.006	NA	NA	NA	NA
Albarakati [28]	Median: 69	IHC	HER2 score ≥ 1.0 in ≥10% tumor cells	55 (27.8)	NA	NA	HR: 2.066, CI 1.262–3.382, *p* = 0.004	HR: 1.955, CI 1.258–3.037, *p* = 0.003	Median: 35.4
Bai [21]	Median: 66	RPPA + RNA-Seq (mRNA)	High HER2 = protein/RPPA above median	172 (50)	HR: 0.970 CI: (0.689–1.367) *p* = 0.864	NA	NA	HR: 0.832, CI 0.550–1.259, *p* = 0.385	Median: 17.6
Chen [15]	Median: 62.4	IHC	≥5% stained cells → 1+ (low) ≥50% stained → 2+ (high)	46 (40.3)	NA	HR: 1.38, CI 0.64–2.95, *p* = 0.41	NA	HR: 1.34, CI 0.58–3.11, *p* = 0.49	Median: 27
Zhou [17]	Median: 64	IHC	IHC 2+ or 3+ (Depend on HercepTest Breast Manual)	51 (53)	in 69 pts with pT2–pT4: HR: 2.501 CI: (1.090–5.743) *p* = 0.031	NA	NA	HR: 2.729, CI 0.076–6.332, *p* = 0.019	NA
Kim [18]	Median: 65.5	IHC	IHC score 2+ or 3+ = overexpression	62 (57.4)	HR: 3.03 CI: (0.95–9.74) *p* = 0.062	NA	NA	HR: 3.61, CI 1.07–12.18, *p* = 0.039	Median: 31.5
Huang [22]	Median: 68	IHC and FISH	HER2-positive = IHC 3+ OR IHC 2+ with FISH amplification HER2-low Definition: IHC 1+ OR IHC 2+ without FISH amplification	16 (21.3)		HR ^a^: 7.877, CI 1.096–56.64, *p* = 0.04	NA	NA	Up to 100

NA: not available, HER2: human epidermal growth factor receptor 2, IHC: immunohistochemistry, FISH: fluorescence in situ hybridization, DISH: dual-hapten in situ hybridization, RPPA: reverse-phase protein array, NGS: next-generation sequencing, CEP17: single copies of chromosome 17, TOP2A: target DNA topoisomerase IIα, pT: pathological T, OS: overall survival, CSS: cancer-specific survival, PFS: progression-free survival, RFS: recurrence-free survival, HR: hazard ratio, CI: confidence interval, RR: risk ratio, pts: patients. a: HER2-high as reference.

## Data Availability

Data derived from public domain resources (The data presented in this study are available in reference number [13,14,15,16,17,18,19,20,21,22,23,24,25,26,27,28,29]).

## Supplementary Materials

The following supporting information can be downloaded at: https://www.mdpi.com/article/10.3390/ijms27094130/s1.

## Abbreviations

The following abbreviations are used in this manuscript:

HER2	Human epidermal growth factor receptor 2
UC	Urothelial carcinoma
PRISMA	Preferred Reporting Items for Systematic Reviews and Meta-Analyses
RFS	Recurrence-free survival
PFS	Progression-free survival
CSS	Cancer-specific survival
OS	Overall survival
IHC	Immunohistochemistry
FISH	Fluorescence in situ hybridization
HR	Hazard ratio
RR	Risk ratio
CI	Confidence interval
